# HPV and HIV Among Youth: Exploring the Role of Knowledge, Risk Perception, and Attitude to Vaccination in Prevention Strategies

**DOI:** 10.3390/vaccines14010101

**Published:** 2026-01-21

**Authors:** Silvia Cocchio, Andrea Cozza, Matilde Obici, Elisabetta Conte, Claudia Cozzolino Cangiano, Nicoletta Parise, Patrizia Furlan, Vincenzo Baldo

**Affiliations:** 1 Department of Cardiac, Thoracic, Vascular Sciences, and Public Health, University of Padua, 35128 Padua, Italy; silvia.cocchio@unipd.it (S.C.); andrea.cozza@unipd.it (A.C.); matilde.obici@studenti.unipd.it (M.O.); elisabetta.conte.2@studenti.unipd.it (E.C.); claudia.cozzolinocangiano@studenti.unipd.it (C.C.C.); patrizia.furlan@unipd.it (P.F.); 2 Preventive Medicine and Risk Assessment Unit, Azienda Ospedale Università Padova, 35128 Padua, Italy; 3 Department of Philosophy, Sociology, Education and Applied Psychology, University of Padua, 35139 Padua, Italy; nicoletta.parise@unipd.it

**Keywords:** prevention, vaccination, risk, knowledge, sexually transmitted infections, HPV, HIV

## Abstract

**Background**: Sexually transmitted infections (STIs) represent a significant public health problem due to their impact. Knowledge about them, perceptions of the risk of contracting them, and adherence to prevention strategies such as HPV vaccination are, at various levels, key factors in preventing the spread of STIs. The study therefore aimed to investigate and evaluate, in a group of young Italians, the level of knowledge, perception of risk and propensity to adhere to preventive strategies, including vaccination against papillomavirus. **Methods**: A cross-sectional study was conducted by administering a questionnaire to young people aged between 16 and 30, residing in four macro-geographical areas, collecting socio-demographic, behavioral and knowledge data. Levels of knowledge about STIs and HPV were classified into four categories (low, medium without awareness, medium with awareness, high). Risk perception was assessed on a scale of 1 to 10. **Results**: A total of 2576 questionnaires were collected, revealing that general knowledge about STIs is limited: only 12.5% of participants demonstrated a high level of knowledge, while 27.1% demonstrated a low level; with regard to HPV, 41.3% of the sample demonstrated a low level of knowledge. The perception of the risk of contracting HIV and HPV was low in most subjects (average score of approximately 2.9 out of 10), with no significant differences related to levels of knowledge about HPV. Potential adherence to HPV vaccination was high (83.0% considered vaccination useful), but among unvaccinated subjects, almost half expressed concerns about vaccination, related to poor knowledge and mistrust of vaccines in general. Factors associated with a higher frequency of self-reported STIs included older age, transgender identity, non-heterosexual orientation, and risky sexual behavior. **Conclusions**: The results emerging from the study highlight the urgent need to strengthen educational and preventive interventions aimed at young people. Raising awareness of the risk of contracting STIs and the importance of vaccination are key targets for health promotion interventions.

## 1. Introduction

One of the major global public health issues is represented by sexually transmitted infections (STIs), which contribute to a high disease burden, significantly impacting the quality of life and public health [1,2].

### 1.1. HPV Infection

Among these, HPV (Human Papillomavirus) infection is one of the most widespread sexually transmitted infections globally, while HIV (Human Immunodeficiency Virus) infection is among the most media-impactful globally [3,4]. Human papillomaviruses are small DNA viruses, with over 100 identified types that infect humans (both sexes), about 40 of which are associated with benign and malignant diseases of the anogenital tract. Different HPV types are categorized based on their oncogenic potential into high- and low-risk types, with at least 12 types demonstrating oncogenic evidence [5]. Some HPV types have a higher tendency to progress into cervical carcinoma than others. It is estimated that HPV 16 and HPV 18 account for over 70% of cervical cancer cases. Low-risk HPV types are associated with benign lesions such as genital warts, with HPV 6 and HPV 11 responsible for over 90% of wart cases. HPV is sexually transmitted through skin or mucosal contact. Microtraumas occurring during sexual intercourse may facilitate transmission. HPV epidemiology highlights a highly prevalent infection among sexually active individuals. According to the World Health Organization (WHO), HPV is responsible for approximately 99% of cervical cancer cases and contributes to over 600,000 new HPV-related cancers each year [6]. A meta-analysis of 78 studies published between 1995 and 2005 estimated an HPV prevalence of 10.4% among women with normal cytology in less developed countries, with prevalence varying by age group, with higher averages among women under 25 years old (16.9%). In women with cytological abnormalities, HPV infection prevalence is higher, particularly in cases of cervical carcinoma and high-grade lesions compared to low-grade lesions [7]. Another meta-analysis of 194 studies published between 1995 and 2009 estimated an HPV prevalence of 11.7% among 1 million women with normal cytology over 5 continents [8]. In Europe, high-risk HPV infection prevalence ranges from 3% to 15% [9].

Regarding HPV knowledge and vaccination, a 2019 study found that 51.8% were knowledgeable about HPV and 75.9% about vaccination, with higher knowledge levels among women and individuals with higher education [10]. Another 2020 study conducted on European adolescents and their parents reported a knowledge rate of 51.8% among adolescents and 64.4% among parents [11]. Vaccination, alongside cervical cancer screening programs, remains the primary preventive strategy. Currently available vaccines protect against the nine most high-risk HPV serotypes, preventing over 90% of virus-related cancers [12]. In Italy, HPV vaccination is recommended and free of charge offered to adolescents, starting at age 11.

### 1.2. HIV Infection

HIV (Human Immunodeficiency Virus), on the other hand, targets CD4 lymphocytes, which play a crucial role in the immune response. Consequently, HIV infection weakens the immune system, increasing vulnerability to other pathogens like viruses, bacteria, protozoa, fungi and cancers [13]. HIV does not cause specific symptoms but manifests through its impact on the immune system. The presence of HIV antibodies in the blood is defined as HIV seropositivity. An HIV-positive individual may live asymptomatically for years, becoming aware of the infection only through an opportunistic disease. AIDS (Acquired Immune Deficiency Syndrome) represents the advanced stage of HIV infection, occurring when CD4 cells decline to a point where the immune system fails to respond to infections. Regarding HIV/AIDS epidemiology, UNAIDS (The Joint United Nations Programme on HIV/AIDS) data estimate that in 2021, 38.4 million people were living with HIV globally, with 1.5 million new diagnoses. Of these, 36.7 million were adults, 1.7 million were children under 15, and 54% were female [14]. In Europe, the “HIV/AIDS Surveillance in Europe 2022” reported 106,508 new HIV diagnoses and 8194 new AIDS cases with an incidence of 1.2 per 100,000 inhabitants [15]. According to the Italian National Institute of Health (Istituto Superiore di Sanità, ISS) report, 2349 new HIV diagnoses were reported in Italy in 2023, with an incidence of four cases per 100,000 residents, and 76% of cases were male. The National AIDS Registry quotes that, from 1982 to date (February 2025), there have been 73,150 AIDS cases and 47,862 deaths up to 2021 [16].

Regarding HIV/AIDS knowledge and behaviors, a 2014 study showed a “moderate” knowledge level but lacked awareness of specific aspects such as TasP (treatment as prevention), the three-month window period for definitive HIV test results, and post-exposure prophylaxis [17]. Despite significant progress in HIV research, no vaccine is yet available to definitively prevent infection.

The aims of this study are to estimate the frequency of self-reported sexually transmitted infections (STIs) in a sample of young Italians and to determine the socio-demographic variables and sexual behaviors associated with them; to investigate the perception of risk of contracting HIV and HPV in relation to STI knowledge levels, sexual behaviors, and socio-demographic variables; to assess the willingness to receive HPV vaccination and profile those who are hesitant.

## 2. Materials and Methods

### 2.1. Study Design

A questionnaire was developed to assess young people’s perspectives on sexuality-related topics. The questionnaire was administered in December 2023 to a statistically representative sample of Italian youth aged 16 to 30, residing in four geographical areas with comparable prevention plans and vaccination policies (North-West, North-East, Center, and South). The questionnaire was an on-line format, and it consisted of 24 questions divided into five sections. In this study, in addition to demographic responses, the analyzed data included self-reported infections, STI diagnoses, general knowledge of STIs and HPV, risk perception of contracting HIV and HPV, and willingness to receive HPV vaccination. The data were collected anonymously.

The questions related to STI and HPV knowledge included 14 and 11 items, respectively. All questions had three possible responses: “True”/“False”/“I don’t know.” Knowledge levels were assessed using a categorical variable that considered both the number of correct answers and the number of “I don’t know” responses. Specifically, the following categories were defined: low level (correct answers ≤ 3/14 for STIs and ≤3/11 for HPV), medium without awareness (correct answers between 4–9 for STIs and 4–7 for HPV, with the number of “I don’t know” responses equal to or lower than the number of incorrect answers), medium with awareness (correct answers between 4–9 for STIs and 4–7 for HPV, with the number of “I don’t know” responses exceeding the number of incorrect answers), and high level (correct answers ≥ 10/14 for STIs and ≥8/11 for HPV).

Contracting HIV and HPV risk perception was evaluated on a scale of 1 to 10 (1 indicates “very low risk” and 10 indicates “very high risk”).

The variable “Educational level” was classified based on “Years of study” (<13 years; 13 years; >13 years). In the sample people with higher educational qualifications are overrepresented, thus a weight was assigned to this variable to ensure that the distribution of respondents by age class, education level, and gender matched the resident population as recorded by ISTAT. This weighting was applied in all analyses.

Non-binary individuals were excluded from the analysis due to their low representation and because ISTAT data do not provide information on this gender category.

### 2.2. Statistical Analysis

The data collected through the questionnaire were presented as means ± standard deviations (SD) for continuous variables and as percentages for categorical variables. Continuous variables were compared using Student’s t-test or ANOVA, while categorical variables were analyzed using the Chi-square test. To investigate which variables were significantly associated with a higher frequency of STI diagnoses, a logistic regression analysis was performed, adjusting for key covariates of interest (socio-demographic variables, risk behaviors, and geographical area). Adjusted odds ratios (adjORs) with 95% confidence intervals were estimated. Linear regressions were conducted to assess how the perception of risk for contracting an HPV or HIV infection (dependent variable) varied as a function of socio-demographic variables, educational attainment, economic status, geographical area, behaviors (oral and anal sex, condom use, have attended a sex education course), and knowledge level (HPV or STIs, respectively). Correlation was estimated using the β coefficient (95%CI) with the corresponding 95% confidence interval. Finally, a logistic regression analysis was performed to profile individuals unfavorable to HPV vaccination, adjusting for socio-demographic variables, risk behaviors, the presence or absence of STI diagnosis, and HPV knowledge level. Adjusted odds ratios (adjORs) with 95% confidence intervals were estimated. A *p*-value < 0.05 was considered statistically significant. Analyses were performed using the Statistical Package for the Social Sciences (SPSS 28.0; SPSS Inc., Chicago, IL, USA).

### 2.3. Ethical Committee Approval

This study was conducted in accordance with the Declaration of Helsinki and was approved by the Ethical Committee (no. 0001419 3 April 2024, University of Padua).

## 3. Results

### 3.1. STI Frequency

The study sample consisted of a total of 2576 individuals. Among them, 51.7% were male (n = 1333), 96.1% (n = 2474) identified with their biological sex (cisgender), 81.5% (n = 2100) identified as heterosexual, and 94.4% (n = 2433) held Italian Nationality. The mean age of the sample was 23.2 ± 3.9 years. Specifically, 22.1% of participants were between 16 and 19 years old, 39.8% were between 20 and 24 years old while 38.1% were between 25 and 30 years old. A total of 9.2% (n = 238) reported having received an STI diagnosis, while 2.5% (n = 64) abstained from answering. The average age of those who reported an STI diagnosis was significantly higher (24.2 ± 4.0 years) compared to those who did not report an STI (23.1 ± 3.9 years) (*p* < 0.001).

Regarding education level, 34.3% of respondents (n = 882) had less than 13 years of education, 49.0% (n = 1262) had 13 years, and 16.7% (n = 431) had more than 13 years. A total of 38.2% (n = 985) were employed, and 43.9% reported having financial difficulties, with 34.3% experiencing some difficulties and 9.6% facing significant financial hardship. In terms of geographical distribution, 39.4% of respondents were from the North, 40.4% from the Center, and 20.2% from the South.

Regarding behaviors, 70.7% of the sample reported engaging in oral sex, 38.5% in anal sex, and 50.1% had attended a sex education course. In the year preceding the survey, 1653 individuals (64.2%) reported being sexually active. Among them, 33.8% (n = 559) consistently used condoms, while 63.5% used them inconsistently, with 16.9% (n = 280) using them often and 46.6% (n = 770) using them occasionally, rarely, or never.

In terms of STI knowledge, 27.1% had a low level, 60.4% had a medium level (28.0% with medium knowledge without awareness and 32.4% with medium knowledge with awareness), and 12.5% had a high level. HPV knowledge was found to be lower than STI knowledge, with 41.3% of the sample having a low level, 46.5% a medium level (23.5% medium without awareness and 22.6% medium with awareness), and 12.2% a high level (Table 1).

The multivariate analysis, shown in Table 2, was conducted on the 2512 individuals who responded to the STI diagnosis question, revealed that the frequency of self-reported STI diagnoses increased with age and specifically it was significantly higher among young people aged 25–30 years compared to those aged 16–19 years (12.0% vs. 7.7%, adjOR (95%CI): 1.78 (1.08–2.91)). It was also significantly higher among transgender individuals (28.5% vs. 8.8%, adjOR (95%CI): 1.88 (1.06–3.34)), non-heterosexual individuals (16.8% vs. 7.9%, adjOR (95%CI): 2.23 (1.56–3.17)), those with less than 13 years of education compared to those with more than 13 years (13.2% vs. 8.0%, adjOR (95%CI): 2.06 (1.28–3.30)), those with fewer financial difficulties (adjOR (95%CI): 2.18 (1.24–3.82)), and those who engaged in oral sex (11.5% vs. 3.2%, adjOR (95%CI): 2.17 (1.23–3.85)) and anal sex (15.6% vs. 4.8%, adjOR (95%CI): 2.39 (1.69–3.39)) (Table 2).

### 3.2. Risk Perception

In response to the question, “If you have not been diagnosed with an STIs, on a scale from 1 to 10, how exposed do you feel today to the risk of contracting an HIV or HPV infection?”, the average score was 2.93 (95%CI: 2.83–3.02) for HIV and 2.91 (95%CI: 2.81–3.00) for HPV.

Regarding HIV risk perception, 46.9% of participants assigned a score of 1, 8.6% assigned a score of 2 and 82.9% assigned a score between 1 and 5 (median: 2; Q1:1; Q3:5).

Similarly, for HPV risk perception, 46.3% of participants assigned a score of 1, 9.2% assigned a score of 2 and 83.5% assigned a score between 1 and 5 (median: 2; Q1:1; Q3:5) (Table 3).

The linear regression analysis, conducted on a sample of 2274 individuals who did not report an STI diagnosis, revealed that HIV risk perception was higher among not heterosexual (β(95%CI): 0.36 (0.08; 0.64)) and among younger people aged 16–19 years (β(95%CI): 0.42 (0.07; 0.76)) and 20–24 years (β(95%CI): 0.55 (0.30; 0.80)) compared to those aged 25–30 years. It was also higher among individuals who practice oral sex (β(95%CI): 0.54 (0.21; 0.86)) and anal sex (β(95%CI): 0.39 (0.15; 0.63)) and among those who always use condoms (β(95%CI): 0.31 (0.02; 0.59)) or often (β(95%CI): 0.49 (0.13; 0.85)), compared to individuals who use it occasionally, sometimes or never. Conversely, HIV risk perception decreases with worsening economic conditions (β(95%CI): −0.27 (−0.48; −0.06)).

Regarding knowledge levels, HIV risk perception is lower among individuals with low STI knowledge (β(95%CI): −0.45 (−0.73; −0.17)) and high STI knowledge (β(95%CI): −0.48 (−0.82; −0.13)), compared to those with intermediate knowledge without awareness (Table 4).

For HPV, risk perception was higher among younger people aged 20–24 years (β(95%CI): 0.40 (0.15; 0.64)) compared to those aged 25–30 years, among individuals engaging in oral sex (β(95%CI): 0.54 (0.22; 0.85)) and anal sex (β(95%CI): 0.46 (0.23; 0.70)), and those who always use condoms (β(95%CI): 0.28 (0.01; 0.56)) or often (β(95%CI): 0.36 (0.01; 0.72)). HPV risk perception also decreases with worsening economic conditions (β(95%CI): −0.22 (−0.42; −0.01)). No correlation emerged between HPV risk perception and HPV knowledge levels (Table 4).

### 3.3. HPV Vaccination Willingness

**Figure 1:**
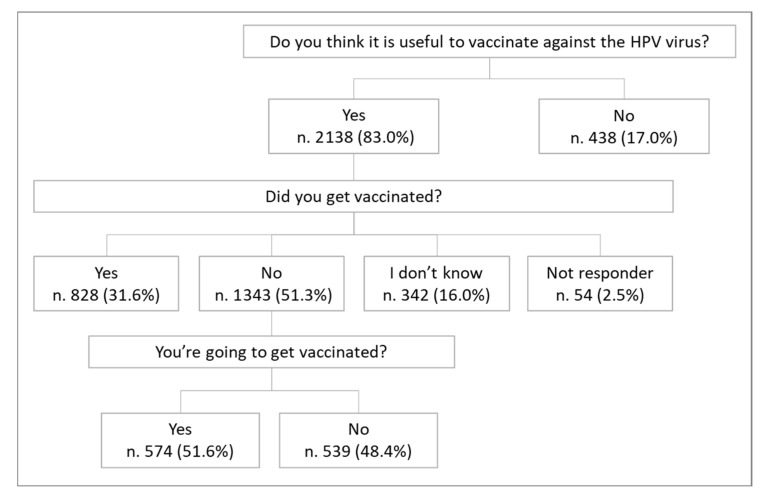
Flow-chart. Propensity for HPV vaccination.

Overall, 83.0% of the sample (n = 2138) considered HPV vaccination useful. Among them, 31.6% (n = 828) were already vaccinated, 51.3% (n = 1343) were not vaccinated, 16.0% (n = 342) were unsure and 2.5% (n = 54) had not responded. Among those who responded that they had not been vaccinated, when asked if they intended to get vaccinated, 539 individuals (48.4%) responded negatively (Figure 1).

**Figure 2:**
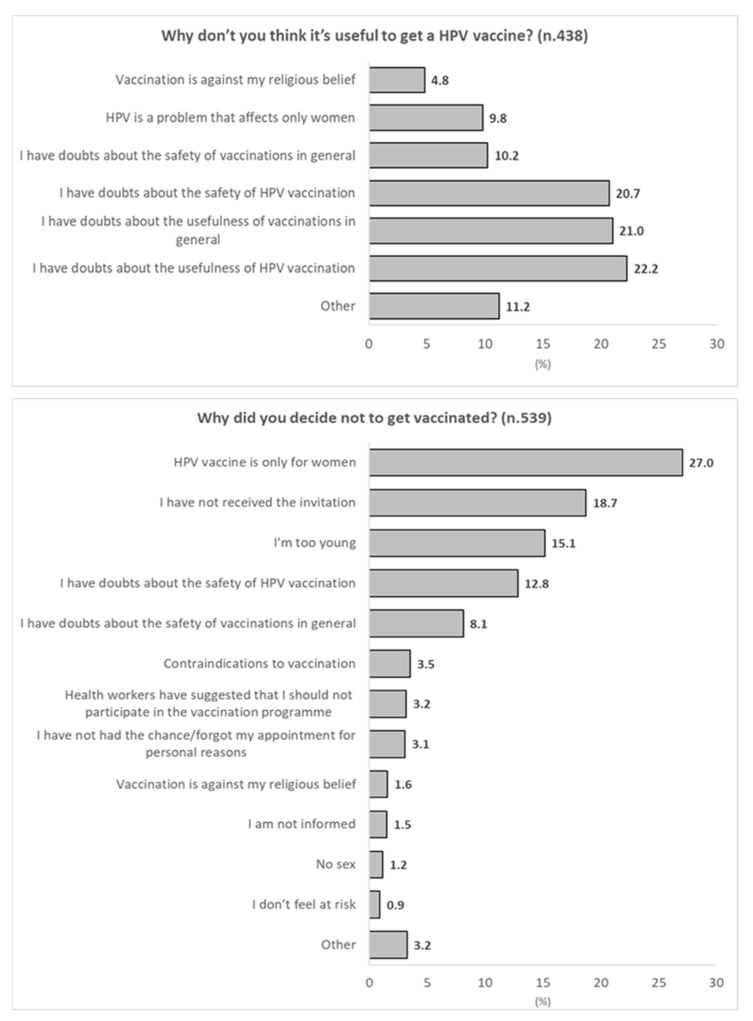
Reasons for considering HPV vaccination unnecessary and for vaccine refusal.

Among those who did not consider HPV vaccination useful, the majority expressed doubts about the usefulness of vaccines in general (21.0%) and concerns about the usefulness (22.2%) and safety (20.7%) of the HPV vaccine specifically. The main reasons for refusing vaccination among the 539 unvaccinated individuals who also did not intend to get vaccinated were the belief that HPV only affects women (27.0%), not having received an invitation (18.7%), considering themselves too young to be vaccinated (15.1%), concerns about vaccine safety in general (8.1%), and specific concerns about the safety of the HPV vaccine (12.8%) (Figure 2).

Among the 2138 individuals who considered HPV vaccination useful, the preferred locations for vaccination were vaccination centers (62.2%), hospitals (20.1%), general practitioners (13.0%), and pharmacies (3.8%), while 1.0% preferred home-based or mobile units near their residence.

Among the 2274 respondents who did not report an STI diagnosis, no significant correlation emerged between HPV risk perception and willingness to receive HPV vaccination.

**Figure 3:**
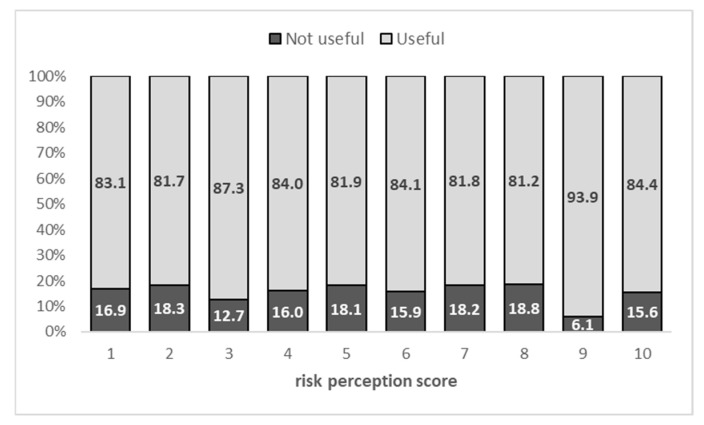
Distribution of subjects in favor and not in favor of HPV vaccination, by HPV risk perception score, among subjects without an STI diagnosis.

In fact, the percentage of individuals in favor of vaccination did not increase as risk perception increased; instead, it remained nearly constant, with an average of 84.3% (Figure 3).

Table 5 illustrates the distribution of respondents who did not consider HPV vaccination useful across the main covariates of interest. The multivariate analysis outlines their profile. The individuals most opposed to HPV vaccination are males (23.0% vs. 10.6%; adjOR (95%CI): 2.64 (2.04–3.42)), foreigners (26.5% vs. 16.5%, adjOR (95%CI): 1.77 (1.13–2.78)), those with lower educational attainment (21.6% vs. 14.8%, adjOR (95%CI): 1.40 (1.05–1.85)) and individuals facing significant economic difficulties (25.1% vs. 16.9%, adjOR (95%CI): 1.79 (1.23–2.61)).

Additionally, skepticism toward vaccination decreases as HPV knowledge levels rise. Specifically, 6.2% of individuals with high HPV knowledge were skeptical about vaccination compared to 22.4% among those with low knowledge levels (adjOR (95%CI): 3.77 (2.20–6.49)).

## 4. Discussion

Overall, 9.2% of the sample, corresponding to approximately one in ten subjects, reported having received an STI diagnosis, specifically about 17% among those who identify as non-heterosexual. A study conducted in 2019 among MSM (Men Who Have Sex with Men) in the Veneto Region recorded a prevalence of 27% [18].

The frequency of STIs seems positively correlated with age. We have some hypotheses about this. The correlation could be linked to more frequent sexual experiences, which potentially increase the risk of contracting infections. Additionally, older individuals may be more attentive to identifying specific symptoms and consequently seek consultation with a specialist. Furthermore, increased awareness of the risks associated with sexual activity and the potential emergence of STIs may encourage individuals to consult healthcare professionals for potential diagnoses.

A higher frequency of STIs is significantly correlated with both lower levels of education and risky sexual behaviors. Data indicate that individuals with fewer than 13 years of education have a higher frequency of self-diagnosed STIs compared to those with a higher level of education. In the literature, the relationship between education level and STI frequency has been widely analyzed [19,20]; however, to establish a direct relationship between these two variables, a more comprehensive analysis is needed, considering additional associated factors such as lifestyle and other individual and socio-cultural factors.

A higher frequency of STIs is significantly correlated with risky sexual behaviors, as highlighted by several studies, including that of Rusley et al. This study reported an increase in STIs among young adults who did not adopt preventive measures such as condom use or had multiple sexual partners [21]. Similarly, the study by Voyiatzaki et al. found that in the sample considered, only 4 out of 10 individuals used condoms during sexual intercourse [22].

The collected data show an overall low level of risk perception for both HIV and HPV. This aspect is supported by scientific literature and may represent one of the main challenges in the field of STIs [23,24]. Risk perception is a subjective aspect influenced by several factors, such as prior knowledge, personal experiences, cultural beliefs and access to health information. In relation to acquired knowledge and the collected and analyzed data, an apparent paradox related to knowledge emerges, and an especially interesting finding appears regarding the relationship between STI knowledge and HIV risk perception. Individuals with low STI knowledge seem to perceive a lower risk compared to those with a medium level of knowledge, which could be explained by a lack of awareness regarding transmission mechanisms [25]. However, even those with high knowledge levels show a reduced risk perception compared to those with intermediate knowledge. This apparent contradiction can be interpreted in two ways. The first one is caused by excessive confidence: those with extensive knowledge of STIs might be overly confident in their protected behaviors and believe they can control risk through individual strategies. The second relevant aspect concerns trust in one’s partner and relationship: one of the main reasons for not using condoms is trust in the partner’s fidelity [26]. This could explain why individuals with high knowledge do not perceive an equally high risk, especially if they are in a stable relationship [24]. Additionally, emotional and psychological experiences also play an important role: the psychological discomfort associated with condom use can lead even those aware of risks to avoid protective measures. The perception of the risk of HIV and HPV is correlated with age, being higher among younger individuals than among those aged 25 to 30 years, who also appear to represent the age group with the highest frequency of self-reported STIs. The perception of the risk of HIV and HPV also decreases with worsening economic conditions [24]. Young people may be more cautious in approaching sexuality and related issues due to excessive caution driven by inexperience. Conversely, having gained experience in sexual matters may lead to a less cautious approach, as these activities are already established and, more likely, due to being in a stable relationship. Economic determinants, ultimately, represent significant limitations in daily life, potentially diverting attention specifically toward financial concerns. This consideration also applies to the frequency of self-diagnosis of STIs.

The risk perception of contracting HIV was found to be higher among non-heterosexual individuals. This data could be linked to the fact that non-heterosexual individuals are aware of being at greater risk for STIs and are also more involved in targeted prevention campaigns regarding sexually transmitted diseases [24].

The risk perception of contracting HPV and HIV was found to be higher among individuals engaging in oral and anal sex. Since these practices are known risk factors, those who practice them may pay greater attention to prevention methods, protective measures, and access to information, thus developing a greater perception of risk than those who do not practice such practices [27].

In this context, data show that a higher perception of the risk of contracting HIV and HPV is associated with more frequent condom use, indicating that risk perception may act as a motivating factor for protective behaviors [28]. However, the absence of a direct correlation between HPV knowledge and risk perception highlights a key issue: available information may not translate into actual behavioral changes or increased personal awareness [29,30]. This underscores the need for educational strategies that go beyond simply providing scientific data, instead working on individual risk perception and psychological barriers to adopting protective behaviors.

Another relevant aspect of the study concerns attitudes toward HPV vaccination, which is characterized by contradictions and cultural resistance [27,31]. In fact, data analysis reveals that even those who consider vaccination useful do not get vaccinated, and there is a low percentage of vaccinated individuals among those who deemed the HPV vaccine useful (31%). This finding suggests that mere belief in the vaccine’s utility is not sufficient motivation, likely due to logistical barriers (difficulty accessing vaccination, lack of sufficient recommendations from healthcare personnel, or excessive costs), procrastination and indecision (even those in favor of vaccination may delay adherence, especially if the vaccine is not mandatory), and concerns about vaccine safety. Those who declare themselves most opposed to vaccination are mainly males, foreigners, individuals with low education levels, and people facing economic difficulties, highlighting an issue of health equity. This is likely because males are often less involved in HPV vaccine awareness campaigns, foreigners and less-educated individuals may have less access to reliable information sources or be influenced by cultural beliefs unfavorable to vaccination, and economic difficulties may lead to perceiving the vaccine as an unnecessary expense, particularly in contexts where vaccination is not free or easily accessible. The correlation between general vaccine skepticism and rejection of HPV vaccination highlights a broader issue of trust—which also regards the relationship between attitudes and scientific literacy in general and awareness campaigns should therefore focus not only on specific HPV information but also on countering vaccine misinformation more broadly [30,32]. Nonetheless, the data reveal a positive aspect: improving disease knowledge and providing clear information about the vaccine’s effectiveness and safety could increase vaccination uptake. Poor disease knowledge is indeed associated with a distorted risk perception, which can negatively influence adherence to preventive measures, including vaccination. Additionally, a lack of understanding of transmission methods and the consequences of contracting the disease leads to underestimating the importance of vaccination. Regarding HPV, vaccination may also be perceived as exclusively linked to sexual activity, which could reduce vaccination adherence.

The study presents several methodological limitations, including potential self-reporting bias and limited sample representativeness. Risk perception may not necessarily translate into protective behaviors, and the influence of various cultural and social factors has not been explored in depth. Finally, it should be considered that the correlation between knowledge and risk perception does not necessarily imply a causal relationship. This study offers some notable strengths that enhance the relevance and robustness of its findings. First, it involved a large and geographically balanced sample of Italian youth, enabling an in-depth analysis of socio-demographic variables and sexual behaviors associated with the risk of sexually transmitted infections (STIs). Second, the questionnaire was designed not only to assess knowledge levels, but also to capture dimensions such as risk perception, behavioral practices, and attitudes toward HPV vaccination—thus providing a comprehensive and multidimensional view of youth sexual health. An additional methodological strength is the use of statistical weighting to adjust for the overrepresentation of individuals with higher educational attainment, thereby improving the generalizability and representativeness of the results. Finally, the study addressed emerging public health challenges, including the paradox between knowledge and risk perception, and identified key barriers to HPV vaccine uptake. These findings offer critical insights to inform more effective and targeted public health strategies.

## 5. Conclusions

The study results highlight a low perception of the risk of contracting sexually transmitted infections, particularly HIV and HPV, among young Italians. This phenomenon is influenced by various factors, including STI knowledge levels, gender, age and socio-economic conditions. What is relevant is the emergence of a knowledge paradox, in which both individuals with a low level of knowledge and those with a high level of knowledge show a lower perception of risk than those with an intermediate level of knowledge. This is consistent with studies that highlighted how the relationship between scientific literacy, attitudes toward science or non-scientific beliefs and behavior is not linear and very complex, so that modifying literacy is not enough to change attitudes and/or behaviors [29,30,33].

Our data suggest the need for targeted educational interventions that go beyond merely providing information and focus on awareness and translating knowledge into protective behaviors. In particular, attention should be focused on young adults (aged 25–30), who have more frequently self-reported STIs but have a lower perception of risk, highlighting a mismatch between real risk and perceived risk.

Regarding HPV vaccination, the study shows that despite a high percentage of individuals being in favor of the vaccine, actual adherence remains low. The main barriers to vaccination include logistical obstacles, misinformation and general vaccine skepticism, with lower uptake among men and individuals with low education levels and economic difficulties, highlighting a disparity in access to prevention.

To raise awareness and improve vaccination rates, interventions involving schools, healthcare workers, and social media could be helpful. Furthermore, it would be desirable to strengthen free and easily accessible vaccination, screening, and counseling services, as well as targeted and differentiated communication strategies capable of reaching specific population groups.

In conclusion, to counteract the spread of HIV and HPV and improve adherence to prevention, a multidisciplinary approach combining health education, equity policies, and effective communication strategies is essential.

## Figures and Tables

**Table 1:** Sample description.

	Total (n.2576)		STI Diagnosis					
			Yes (n.238)		No (n.2274)		nr (n.64)	
	n	(%)	n	(%)	n	(%)	n	(%)
**Total**	**2576**	**(100.0)**	**238**	**(100.0)**	**2274**	**(100.0)**	**64**	**(100.0)**
**Biological Gender**								
*Male*	1333	(51.7)	125	(52.7)	1173	(51.6)	34	(53.3)
*Female*	1244	(48.3)	113	(47.5)	1101	(48.4)	30	(46.2)
**Gender identity**								
*Cisgender*	2474	(96.1)	212	(89.2)	2209	(97.1)	53	(83.2)
*Transgender*	102	(3.9)	26	(11.0)	65	(2.9)	10	(16.2)
**Sexual orientation**								
*Heterosexual*	2100	(81.5)	163	(68.4)	1899	(83.5)	38	(59.7)
*not Heterosexual*	476	(18.5)	76	(31.8)	375	(16.5)	25	(39.8)
**Age (average ± SD)**	23.2 ± 3.9		24.2 ± 4.0		23.1 ± 3.9		22.5 ± 4.0	
**Age group**								
*16–19 years*	570	(22.1)	42	(17.6)	507	(22.3)	21	(32.8)
*20–24 years*	1025	(39.8)	81	(34.0)	922	(40.5)	21	(32.8)
*25–30 years*	981	(38.1)	115	(48.3)	844	(37.1)	22	(34.4)
**Citizenship**								
*Italian*	2433	(94.4)	217	(91.1)	2162	(95.1)	54	(84.3)
*Foreign*	143	(5.6)	22	(9.1)	112	(4.9)	10	(15.2)
**Years of study**								
*<13 ys*	882	(34.3)	113	(47.4)	742	(32.6)	28	(43.5)
*13 ys*	1262	(49.0)	92	(38.8)	1148	(50.5)	22	(33.8)
*>13 ys*	431	(16.7)	33	(14.0)	384	(16.9)	14	(22.1)
**Employment status**								
*Employed*	985	(38.2)	104	(43.7)	859	(37.8)	22	(33.8)
*Looking for employment*	359	(13.9)	39	(16.6)	302	(13.3)	17	(27.3)
*not in labor force*	1232	(47.8)	95	(39.9)	1113	(48.9)	25	(38.4)
**Economic condition at the end of month**								
*Very easy*	352	(13.7)	64	(26.8)	277	(12.2)	11	(16.8)
*Quite easy*	1093	(42.4)	84	(35.3)	989	(43.5)	20	(31.6)
*Some difficulties*	884	(34.3)	64	(26.9)	796	(35.0)	24	(37.8)
*Many difficulties*	247	(9.6)	26	(11.1)	213	(9.3)	8	(13.2)
**Geographical area**								
*North-West*	518	(20.1)	51	(21.5)	450	(19.8)	18	(27.4)
*North-East*	497	(19.3)	44	(18.3)	446	(19.6)	8	(12.1)
*Center*	1040	(40.4)	101	(42.6)	912	(40.1)	26	(41.0)
*South*	521	(20.2)	42	(17.7)	466	(20.5)	12	(19.0)
**Oral sex**								
*yes*	1821	(70.7)	205	(86.2)	1579	(69.4)	37	(58.5)
*no/nr*	755	(29.3)	33	(13.9)	695	(30.6)	27	(42.2)
**Anal sex**								
*yes*	991	(38.5)	151	(63.4)	815	(35.8)	25	(39.1)
*no/nr*	1585	(61.5)	87	(36.6)	1459	(64.2)	39	(60.9)
**Condom use in the last year**								
*Always*	559	(21.7)	55	(23.0)	494	(21.7)	11	(17.3)
*Often*	280	(10.9)	38	(16.0)	233	(10.2)	9	(13.8)
*Occasionally/sometimes/never*	770	(29.9)	100	(42.1)	659	(29.0)	11	(16.7)
*No sex/nr*	967	(37.5)	45	(19.0)	889	(39.1)	33	(51.6)
**Sexuality education**								
*yes*	1291	(50.1)	149	(62.7)	1112	(48.9)	30	(46.5)
*no*	1285	(49.9)	89	(37.5)	1162	(51.1)	34	(53.0)
**STI Knowledge**								
*Low*	699	(27.1)	38	(16.1)	633	(27.8)	28	(43.5)
*Medium without awareness*	721	(28.0)	101	(42.5)	596	(26.2)	24	(38.1)
*Medium with awareness*	834	(32.4)	57	(23.8)	770	(33.9)	7	(11.5)
*High*	321	(12.5)	42	(17.7)	275	(12.1)	4	(6.3)
**HPV Knowledge**								
*Low*	1064	(41.3)	73	(30.5)	952	(41.9)	39	(61.4)
*Medium without awareness*	615	(23.9)	104	(43.7)	494	(21.7)	17	(27.0)
*Medium with awareness*	582	(22.6)	29	(12.2)	547	(24.0)	6	(8.9)
*High*	315	(12.2)	33	(13.9)	281	(12.4)	1	(2.2)

nr: non-responder.

**Table 2:** Distribution of STI diagnoses—Logistic regression.

	Total (n.2512)		STIs (n.238)		adjOR (95%CI)	*p*-Value
	n	(%)	n	(%)		
**Total**	**2512**	**(100.0)**	**238**	**(9.5)**	**--**	
**Biological Gender**						
*Male*	1298	(51.7)	125	(9.7)	0.83 (0.61–1.13)	0.227
*Female*	1214	(48.3)	113	(9.3)	ref	
**Gender identity**						
*Cisgender*	2421	(96.4)	212	(8.8)	ref	
*Transgender*	91	(3.6)	26	(28.5)	1.88 (1.06–3.34)	0.030
**Sexual orientation**						
*Heterosexual*	2062	(82.1)	163	(7.9)	ref	
*not Heterosexual*	450	(17.9)	76	(16.8)	2.23 (1.56–3.17)	0.000
**Age group**						
*16–19 years*	550	(21.9)	42	(7.7)	ref	
*20–24 years*	1004	(40.0)	81	(8.1)	1.34 (0.83–2.17)	0.230
*25–30 years*	959	(38.2)	115	(12.0)	1.78 (1.08–2.91)	0.023
**Citizenship**						
*Italian*	2379	(94.7)	217	(9.1)	ref	
*Foreign*	134	(5.3)	22	(16.3)	1.21 (0.68–2.13)	0.515
**Years of study**						
*<13 ys*	855	(34.0)	113	(13.2)	2.06 (1.28–3.30)	0.003
*13 ys*	1241	(49.4)	92	(7.4)	1.03 (0.66–1.61)	0.892
*>13 ys*	417	(16.6)	33	(8.0)	ref	
**Employment status**						
*Employed*	963	(38.3)	104	(10.8)	1.05 (0.72–1.53)	0.791
*Looking for employment*	342	(13.6)	39	(11.5)	1.19 (0.76–1.86)	0.454
*not in labor force*	1208	(48.1)	95	(7.9)	ref	
**Economic condition at the end of month**						
*Very*	341	(13.6)	64	(18.7)	2.18 (1.24–3.82)	0.007
*Quite*	1073	(42.7)	84	(7.8)	0.86 (0.51–1.45)	0.562
*Some difficulties*	860	(34.2)	64	(7.5)	0.77 (0.45–1.32)	0.343
*Many difficulties*	239	(9.5)	26	(11.0)	ref	
**Geographical area**						
*North-West*	501	(19.9)	51	(10.2)	1.06 (0.66–1.72)	0.799
*North-East*	489	(19.5)	44	(8.9)	0.95 (0.57–1.57)	0.830
*Center*	1014	(40.4)	101	(10.0)	1.22 (0.80–1.85)	0.366
*South*	508	(20.2)	42	(8.3)	ref	
**Oral sex**						
*yes*	1784	(71.0)	205	(11.5)	2.17 (1.23–3.85)	0.008
*no*	520	(20.7)	17	(3.2)	ref	
**Anal sex**						
*yes*	966	(38.5)	151	(15.6)	2.39 (1.69–3.39)	<0.001
*no*	1363	(54.3)	66	(4.8)	ref	

ref: reference; multivariate analysis performed excluding missing data (nr).

**Table 3:** HIV and HPV risk perception score among subjects without STI diagnosis.

Score	HIV		HPV	
	n	(%)	n	(%)
1	1066	(46.9)	1052	(46.3)
2	196	(8.6)	210	(9.2)
3	232	(10.2)	226	(10.0)
4	195	(8.6)	204	(9.0)
5	196	(8.6)	206	(9.0)
6	154	(6.8)	170	(7.5)
7	125	(5.5)	104	(4.6)
8	63	(2.8)	63	(2.8)
9	29	(1.3)	23	(1.0)
10	17	(0.8)	16	(0.7)
**Total**	**2274**	**(100.0)**	**2274**	**(100.0)**
**Distribution**	Mean: 2.93		Mean: 2.91	
	Median: 2		Median: 2	
	Q1:1		Q1:1	
	Q3:5		Q3:5	

**Table 4:** HIV and HPV risk perception (score from 1 to 10)—Linear regression (n.2274, subjects without STI diagnosis).

**Perception of HIV Risk**	**β**	**(95%CI)**	***p*** **-Value**
(model constant)	2.24	(1.68; 2.80)	0.000
Biological gender (M vs. F)	0.19	(−0.02; 0.39)	0.080
Gender identity (transgender vs. cisgender)	−0.05	(−0.65; 0.56)	0.878
Sexual orientation (Not hetero vs. hetero)	0.36	(0.08; 0.64)	0.013
Age 16–19 ys (vs. 25–30 ys)	0.42	(0.07; 0.76)	0.018
Age 20–24 ys (vs. 25–30 ys)	0.55	(0.30; 0.80)	<0.001
<13 ys study (vs. >13 ys)	0.16	(−0.19; 0.50)	0.371
13 ys study (vs. >13 ys)	−0.08	(−0.37; 0.20)	0.571
Citizenship (foreign vs. Italian)	−0.18	(−0.65; 0.28)	0.437
Economic difficulties * (yes vs. no)	−0.27	(−0.48; −0.06)	0.010
Looking for employment (vs. employed)	−0.09	(−0.42; 0.25)	0.611
not in labor force (vs. employed)	−0.11	(−0.37; 0.14)	0.385
Oral sex (yes vs. no)	0.54	(0.21; 0.86)	0.001
Anal sex (yes vs. no)	0.39	(0.15; 0.63)	0.001
Sexuality education (yes vs. no)	−0.03	(−0.23; 0.17)	0.769
STI Knowledge (vs. Medium without awareness)			
*Low*	−0.45	(−0.73; −0.17)	0.002
*Medium with awareness*	−0.16	(−0.41; 0.10)	0.233
*High*	−0.48	(−0.82; −0.13)	0.006
Condom (vs. Occasionally/sometimes/never)			
*Always*	0.31	(0.02; 0.59)	0.035
*Often*	0.49	(0.13; 0.85)	0.008
*No sex*	0.22	(−0.11; 0.54)	0.191
Geographical area (vs. South)			
*North-West*	−0.16	(−0.48; 0.17)	0.349
*North-East*	0.01	(−0.31; 0.33)	0.942
*Center*	−0.16	(−0.44; 0.12)	0.252
**Perception of HPV risk**	**β**	**(95%CI)**	***p*** **-value**
(model constant)	2.25	(1.69; 2.81)	0.000
Biological gender (M vs. F)	0.11	(−0.10; 0.32)	0.300
Gender identity (transgender vs. cisgender)	0.39	(−0.20; 0.99)	0.195
Sexual orientation (Not hetero vs. hetero)	0.27	(0.00; 0.55)	0.054
Age 16–19 ys (vs. 25–30 ys)	0.18	(−0.16; 0.52)	0.291
Age 20–24 ys (vs. 25–30 ys)	0.40	(0.15; 0.64)	0.002
<13 ys study (vs. >13 ys)	0.02	(−0.32; 0.35)	0.922
13 ys study (vs. >13 ys)	−0.15	(−0.43; 0.13)	0.293
Citizenship (foreign vs. Italian)	−0.02	(−0.47; 0.44)	0.946
Economic difficulties * (yes vs. no)	−0.22	(−0.42; −0.01)	0.037
Looking for employment (vs. employed)	−0.06	(−0.39; 0.27)	0.732
not in labor force (vs. employed)	−0.15	(−0.40; 0.11)	0.254
Oral sex (yes vs. no)	0.54	(0.22; 0.85)	0.001
Anal sex (yes vs. no)	0.46	(0.23; 0.70)	0.000
Sexuality education (yes vs. no)	−0.04	(−0.24; 0.16)	0.675
HPV Knowledge (vs. Medium without awareness)			
*Low*	−0.11	(−0.37; 0.15)	0.408
*Medium with awareness*	−0.17	(−0.46; 0.11)	0.235
*High*	−0.03	(−0.37; 0.32)	0.886
Condom (vs. Occasionally/sometimes/never)			
*Always*	0.28	(0.01; 0.56)	0.047
*Often*	0.36	(0.01; 0.72)	0.047
*No sex*	0.34	(0.02; 0.66)	0.037
Geographical area (vs. South)			
*North-West*	−0.16	(−0.48; 0.17)	0.342
*North-East*	−0.03	(−0.35; 0.29)	0.849
*Center*	−0.15	(−0.42; 0.13)	0.290

Multivariate analysis performed excluding missing data (nr). * Economic difficulties (yes: some/many difficulties at the end of month; no: very/quite easy at the end of month)

**Table 5:** Distribution of respondents not favorable to HPV vaccination—Logistic regression.

	Total		Not in Favor of HPV Vaccination			
	n	(%)	n	(%)	adjOR (95%CI)	*p*-Value
**Total**	**2576**	**(100.0)**	**438**	**(17.0)**		
**Biological gender**						
*Male*	1333	(51.7)	307	(23.0)	2.64 (2.04–3.42)	<0.001
*Female*	1244	(48.3)	131	(10.6)	ref	
**Gender identity**						
*Cisgender*	2474	(96.1)	413	(16.7)	ref	
*Transgender*	102	(3.9)	26	(25.4)	1.17 (0.67–2.06)	0.585
**Sexual orientation**						
*Heterosexual*	2100	(81.5)	350	(16.7)	ref	
*not Heterosexual*	476	(18.5)	88	(18.5)	1.21 (0.87–1.67)	0.250
**Age group**						
*16–19 years*	570	(22.1)	108	(18.9)	ref	
*20–24 years*	1025	(39.8)	157	(15.3)	0.94 (0.66–1.34)	0.747
*25–30 years*	981	(38.1)	173	(17.7)	1.24 (0.85–1.82)	0.271
**Citizenship**						
*Italian*	2433	(94.4)	400	(16.5)	ref	
*Foreign*	143	(5.6)	38	(26.5)	1.77 (1.13–2.78)	0.013
**Years of study**						
*<13 ys*	882	(34.3)	191	(21.6)	1.40 (1.05–1.85)	0.020
*13 ys*	1262	(49.0)	187	(14.8)	ref	
*>13 ys*	431	(16.7)	61	(14.1)	1.07 (0.75–1.54)	0.700
**Employment status**						
*Employed*	985	(38.2)	181	(18.4)	1.1 (0.82–1.48)	0.536
*Looking for employment*	359	(13.9)	65	(18.2)	0.98 (0.68–1.42)	0.922
*Not in labor force*	1232	(47.8)	192	(15.6)	ref	
**Economic conditions at the end of month**						
*very/quite easy*	1445	(56.1)	243	(16.9)	ref	
*some difficulties*	884	(34.3)	133	(15.0)	0.89 (0.68–1.17)	0.415
*many difficulties*	247	(9.6)	62	(25.1)	1.79 (1.23–2.61)	0.002
**Oral sex**						
*yes*	1821	(70.7)	292	(16.0)	0.96 (0.69–1.33)	0.808
*no*	527	(20.5)	92	(17.5)	ref	
**Anal sex**						
*yes*	991	(38.5)	179	(18.0)	1.06 (0.81–1.4)	0.659
*no*	1382	(53.6)	207	(14.9)	ref	
**STI diagnosis**						
*yes*	238	(9.3)	41	(17.3)	0.88 (0.59–1.33)	0.549
*no*	2274	(88.3)	377	(16.6)	ref	
**Level of HPV knowledge**						
*Low*	1064	(41.3)	238	(22.4)	3.77 (2.20–6.49)	<0.001
*Medium without awareness*	615	(23.9)	109	(17.8)	3.44 (1.96–6.03)	<0.001
*Medium with awareness*	582	(22.6)	71	(12.2)	2.05 (1.15–3.67)	0.016
*High*	315	(12.2)	20	(6.2)	ref	
**Geographical area**						
*North-West*	518	(20.1)	101	(19.6)	1.24 (0.86–1.78)	0.244
*North-East*	497	(19.3)	76	(15.3)	0.77 (0.52–1.14)	0.195
*Center*	1040	(40.4)	170	(16.4)	0.94 (0.68–1.3)	0.699
*South*	521	(20.2)	91	(17.5)	ref	

ref: reference; multivariate analysis performed excluding missing data (nr).

## Data Availability

The data are available from the corresponding author upon reasonable request.
